# Reproductive health service utilization and associated factors among adolescents (15–19 years old) in Gondar town, Northwest Ethiopia

**DOI:** 10.1186/1472-6963-13-294

**Published:** 2013-08-03

**Authors:** Senafikish Amsalu Feleke, Digsu Negese Koye, Amsalu Feleke Demssie, Zelalem Birhanu Mengesha

**Affiliations:** 1 Department of Nursing, University of Gondar, Gondar, Ethiopia; 2 Department of Epidemiology and Biostatistics, Institute of Public Health, University of Gondar, Gondar, Ethiopia; 3 Department of Health Management and Health Economics, Institute of Public Health, University of Gondar, Gondar, Ethiopia; 4 Department of Reproductive Health, Institute of Public Health, University of Gondar, Gondar, Ethiopia

**Keywords:** Reproductive health, Service utilization, Adolescent, Northwest Ethiopia

## Abstract

**Background:**

The utilization of reproductive health services is an important component in preventing adolescents from different sexual and reproductive health problems. It plays a vital role in safeguarding youth in Sub-Saharan African countries including Ethiopia, which accounts for a high proportion of the region’s new HIV infections as well as maternal and infant mortality ratios. Due to this, assessing adolescent reproductive health service utilization and associated factors has its own contribution in achieving the national Millennium Development Goals (MDG), especially goals 4 to 6.

**Methods:**

A community based cross-sectional study was conducted from April 5–19, 2012, in 4 randomly selected administrative areas of Gondar town. A total of 1290 adolescents aged 15–19 were interviewed using a pre-tested and structured questionnaire. Data were entered in to the EPI INFO version 3.5.3 statistical software and analyzed using an adapted SPSS version 20 software package. Logistic regression was done to identify possible factors associated with family planning (FP), and voluntary counseling and testing (VCT) service utilization.

**Results:**

Out of the total participants, 79.5% and 72.2% utilized FP and VCT services, respectively. In addition, among sexually experienced adolescents, 68.1% and 88.4% utilized contraceptive methods and VCT service during their first sexual encounter, respectively. Educational status, discussion with family/relatives, peer groups, sexual partners and teachers were significantly associated with FP service utilization. Also, adolescents who had a romantic sexual relationship, and those whose last sexual relationship was long-term, were about 6.5 times (Adjusted Odds Ratio [AOR] = 6.5, 95% CI: 1.23, 34.59), and about 3 times (AOR = 3, 95% CI: 1.02, 8.24) more likely to utilize FP services than adolescents who had no romantic relationship or long-term sexual relationship, respectively. In addition, the variables significantly associated with VCT service utilization were: participants who had secondary education and above, schooling attendance, co- residence with both parents, parental communication, discussion of services with peer groups, health workers, and perception of a risk of HIV/AIDS.

**Conclusions:**

The majority of the adolescents were utilizing FP and VCT service in Northwest Ethiopia. But among the sexually experienced adolescents, utilization of FP at first sexual intercourse and VCT service were found to be low. Educational status, schooling attendance, discussion of services, type of sexual relationship and perception of risk were important factors affecting the utilization of FP and VCT services. Building life skill, facilitating parent to child communication, establishing and strengthening of youth centers and school reproductive health clubs are important steps to improve adolescents’ reproductive health (RH) service utilization.

## Background

Reproductive health care is defined as the constellation of methods, techniques and services that contribute to reproductive health and well-being by preventing and solving sexual health problems [1]. Globally, the adolescent population is estimated to be 1.25 billion [2]. Among these, 513 million are between 15–19 years old [3], and 85% of the total adolescents are living in developing countries [4]. These adolescents are most vulnerable to a range of reproductive health problems, such as teenage pregnancy and childbearing, unsafe abortion and sexually transmitted infections (STI), including HIV [5].

The World Health Organization (WHO) estimates that in Africa, 60% of all new HIV- infections occur in adolescents who are 15–19 years old [6]. In developing countries, there are about 12.8 million births by adolescents aged between 15–19 years, and a large proportion of these pregnancies are unplanned [7]. Among married women who are 15–19 years old, only 17 percent practice family planning methods currently, and among unmarried sexually active adolescents, the use of contraception is believed to be even lower [3]. A WHO report in Algeria, Bangladesh, Ethiopia, Indonesia and Nigeria showed that the risk of dying from complications related to pregnancy or childbirth is two times higher for those aged 15–19 than for women in their mid-twenties [3].

According to the 2011 Ethiopian Demographic and Health Survey, contraceptive use among currently married women of 15–19 years of age was only 23%, with 0% utilization of permanent methods, 1.6% and 2.5% utilization of implants and IUD, respectively. Contraceptive use was lower when compared with other age groups. Among sexually active youth aged 15–19 years, women and men who were tested for HIV test were only 24% and 27% respectively [8]. Therefore, assessing factors affecting reproductive health service utilization within this age category, especially FP and VCT services, are very important to improve adolescent reproductive health service utilization and thereby reduce the burden of adolescent disease and disabilities associated with reproductive health.

## Methods

### Study design and set up

A community based cross-sectional study was conducted in Gondar town, Northwest Ethiopia from April 5–19, 2012. Gondar town is found in North Gondar Zone of the Amhara Regional State, 750 km Northwest of Addis Ababa. According to the 2007 Ethiopian census report, Gondar had a total population of 206,987, and adolescents aged 15–19 years were estimated to be 12% (25,128) of the total population [9]. The town is divided into 12 administrative areas. The participants of this study were adolescents 15–19 years old who had lived in the area for at least six months.

### Sample size and sampling procedure

The sample size was determined by using the single population proportion formula with the following assumptions: 17.6% proportion [10], 95% confidence level, 3% margin of error and design effect of 2. Then five percent was added for the expected non-response, making the final sample size 1300. From the 12 administrative areas of Gondar town, 4 were randomly selected. The sample allocated was proportional to the household size of each administrative area. The first household from each administrative area was identified using the lottery method, and then the systematic random sampling technique was applied to identify the next household to be included. Adolescents who were found in the selected households were interviewed. In the case of more than one eligible participant in the household, the lottery method was used to select one. In households in which adolescents were not at home, but it was known that there were eligible adolescents for the study, the interviewers revisited the household at three different time intervals, and when interviewers failed to meet the adolescent, the household was excluded from the survey and replaced by the next household in a clockwise direction.

### Data collection tool and procedure

A structured questionnaire was developed and administered to the participants. The questionnaire and consent documents were first developed in English, then translated into Amharic, and finally retranslated into English by another translator to check consistency. Before the actual work, data collectors and supervisors were given intensive training for two days about the aim of the study, procedures and data collection techniques by going through the questionnaire question by question. After the training, interviewers pre-tested the questionnaire on 78 (6%) people living in an administrative area not selected for the study.

The following operational definitions were used: Reproductive health services particularly considered in this study were FP and VCT services. FP service utilization was defined as ever use of any modern contraceptives in life time, and VCT service utilization referred to ever utilization of VCT service for HIV testing. Duration of sexual relationship was defined as number of months from first sexual intercourse to the last sexual intercourse. Type of sexual relationship reflects whether adolescents ever had a romantic relationship in their sexual experience or not.

### Data processing and analysis

Data were checked for completeness and entered into EPI INFO version 3.5.3 statistical software and then exported to SPSS version 20 for further analysis. Multiple logistic regression was used to identify variables independently associated with service utilization. The strength of association was interpreted using the adjusted odds ratio and 95% CI. The criterion for statistical significance was set at a p value of 0.05.

### Ethical considerations

Ethical clearance was obtained from the Institution Review Board of the Institute of Public Health, the University of Gondar. Formal letters of cooperation were written to different kebele administrators. Oral consent was obtained either from parents or surrogate and participants. Moreover, the adolescents were assured that neither the interviewer nor their parents would have access to their responses. Confidentiality was assured by using anonymous questions and by conducting the interviews privately throughout.

## Results

### Socio-demographic characteristics of study the participants

Out of 1300 study samples, 1290 responded to our interview making the response rate 99.23%. About half of them (50.4%) were females and 1209 (93.7%) were single. The mean age of the participants was 16.96 years with a standard deviation of 1.4 years. More than three-fourths of the participants (85.5%) were enrolled in school and 647 (50.2%) had at least secondary education. More than one third (44.2%) of the participants’ mothers’ age was between 40–49 years and 699(55.2) of them had no formal education. Around 61.7% were living with both parents (Table 1).

**Table 1:** **Percentage distribution of the study population by socio**-**demographic characteristics**, **Gondar town**, **Northwest Ethiopia**, **April 2012**

**Variables**	**Frequency**	**Percent**
**Sex**		
Male	640	49.6
Female	650	50.4
**Age**		
15–16	536	41.6
17-19	754	58.4
**Marital status**		
Single	1209	93.7
Married	72	5.6
Divorced	9	0.7
**Religion**		
Orthodox	1135	88.0
Muslim	147	11.4
Catholic	2	0.2
Protestant	6	0.5
**Schooling status**		
In school	1103	85.5
Out of school	187	14.5
**Educational status**		
No formal education	67	5.2
Primary education	539	41.8
Secondary education & above	684	53.0
**Age of Mother**		
30-39	444	35
40-49	560	44.2
50-59	210	16.6
60+	53	4.2
**Mother’s Educational status**		
No formal education	699	55.2
Primary education	313	24.7
Secondary education & above	255	20.1
**Co residence with both parents**		
Yes	796	61.7
No	494	38.3

### Sexual history of the adolescents

Out of the total participants, 445 (34.5%) have had sexual partners, and 303 (23.5%) have had sexual intercourse. Out of those who had sexual partners, the majority (84.5%) had one sexual partner. Among those who had sexual intercourse, 294 (97%) had sexual intercourse in the past 12 months and 223 (73.6%) had sexual intercourse more than once with the same sexual partner. Out of those who had sexual intercourse, 269 (88.8%) had a romantic sexual relationship, and 218 (71.9%) stayed in their last sexual relationship for more than six months, with a median duration of 12 months (Table 2).

**Table 2:** **Percentage distribution of the study population by their sexual history**, **Gondar town**, **Northwest Ethiopia**, **April 2012**

**Variables**	**Frequency**	**Percent**
**Ever had Sexual partner**		
Yes	445	34.5
No	845	65.5
**Number of sexual partner**	**n=445**	
One	376	84.5
Two and more	69	15.5
**Ever had sexual intercourse**		
Yes	303	23.5
No	987	76.5
**Ever had sexual intercourse within the last 12 months**	**n=303**	
Yes	294	97
No	9	3
**Amount of sexual intercourse**	**n=303**	
Once	48	15.8
More than once with the same partner	223	73.6
More than once with different partner	32	10.6
**Romantic relationship**	**n=303**	
Yes	269	88.8
No	34	11.2
**Duration of last sexual relationship**	**n=303**	
1-6 months	85	28.1
>6 months	218	71.9

### Family planning service utilization

About 1205 (93.4%) of the participants had awareness about family planning services. One thousand sixteen (82%) of the participants had discussed the service. The majority of the participants, 899 (88.5%), discussed the service with their peers and 667 (65.6%) with their teachers.

Out of those who had sexual intercourse, 241 (79.5%) utilized family planning services. One hundred sixty-four (68.1%) of these used contraceptive methods at their first sexual intercourse, and 234 (97.1%) used contraception at their last intercourse. The most commonly used contraceptive method was the condom, 112 (46.5%), followed by injectables, 103 (42.73%). More users went to health centers, 127 (52.7%), while others, 103 (42.7%), preferred shops for the service. The major reasons for not using family planning services for 57 (87.1%) of the participants was fear of being detected. Others, 37 (59.7%) reported that they disliked the judgmental attitude of health workers.

### Voluntary counseling and testing service utilization

Out of the total participants, 1265 (98%) had heard about the VCT service. The majority of the participants (89%) had discussed the service with someone. Peer groups/friends, 1049 (91.4%), followed by teachers, 771 (67.2%), were the main people with whom the participants of the study discussed VCT services. One fourth of the participants (25%) perceived themselves as being at risk to contracting HIV/AIDS. Out of all adolescents, 932 (72.2%) utilized the VCT service, and out of the sexually experienced, 268 (88.4%) utilized the service. More than half (55.6%) were females, and 608 (65.3%) obtained the service from health centers and 220 (23.61%) from schools. Partner or self trust, 326 (91.1%), and embarrassment, 226 (63.1%), were the main reasons for participants for not using the VCT service.

### Factors associated with family panning service utilization

Based on the bivariate analysis, the factors found to be significantly associated with family planning service utilization were age, schooling attendance, educational status, mother’s educational status, co- residence with both parents, parental monitoring, parental communication, discussion on family planning services, number of sexual partners, amount of sexual experience, having a romantic sexual relationship and duration of last sexual relationship.

Out of variables which were entered to multiple logistic regression, participants’ educational status, maternal educational status, discussion on FP use with family/relatives, peer group, sexual partners and teachers, having romantic and long term sexual relationships were found to be significantly associated with family planning service utilization.

Adolescents with secondary education and above were about 9 times more likely to utilize FP service as compared to those with no formal education (AOR = 9, 95% CI: 1.45, 54.14). Similarly, maternal education was found to have an association with the utilization of family planning services (AOR = 6.4, 95% CI: 1.39, 29.72).

Having romantic sexual relationships and a long duration of the last sexual relationship were also found to be strong predictors of FP service utilization. Adolescents who had romantic sexual relationships were about 6.5 times (AOR = 6.5, 95% CI: 1.23, 34.59) more likely to utilize family planning services than those who had not had romantic sexual relationships. In addition, adolescents who had a long duration of the last sexual relationship were about 3 times (AOR = 3, 95% CI: 1.02, 8.24) more likely to utilize the service than those had a short duration of last sexual relationship.

Discussion with family/relatives, peer group/friends, sexual partners and teachers on family planning were also the other factors significantly and independently associated with the utilization of the service (Table 3).

**Table 3:** **Bivariate and Multivariate analysis of factors associated with family planning service utilization among adolescents**, **Gondar town**, **Northwest Ethiopia**, **April 2012** (**n**=**303**)

**Variables**	**FP service utilization**		**Crude OR (95% CI)**	**Adjusted OR (95% CI)**
	**Yes**	**No**		
**Age**				
15-16	16	19	1	*
17-19	225	43	6.21 (2.96, 13.03)	
**Schooling status**				
In school	173	27	3.29 (1.89, 5.89)	*
Out of school	68	35	1	
**Educational status**				
No	13	25	1	1
Primary	48	18	5.13 (2.17, 12.14)	2 (0.33, 13.12)
Secondary	180	19	18.22 (8.02, 41.4)	9 (1.45, 54.14)
**Maternal educational status**				
No formal education	110	46	1	1
Primary education	79	11	3 (1.46, 6.16)	2.4 (0.75, 7.52)
Secondary education and above	49	5	4.1 (1.53, 10.95)	6.4 (1.39, 29.72)
**Co residence with both parents**				
Yes	119	38	1	*
No	122	24	1.6 (0.98, 2.87)	
**Parental monitoring**				
High	18	8	1	*
Low	223	54	1.84 (0.76, 4.44)	
**Parental communication**				
Yes	118	10	4.49 (2.42, 10.27)	*
No	123	52	1	
**Ever discussed about the service with**				
**Family/relatives**				
Yes	129	15	2.24 (1.13, 4.45)	3 (1.16, 9.37)
No	100	26	1	1
**Peer group/friends**				
Yes	208	26	5.7 (2.63, 12.44)	20 (5.89, 66.18)
No	21	15	1	1
**Sexual partner**				
Yes	181	12	9.11 (4.33, 19.18)	3 (1.12, 8.47)
No	48	29	1	1
**Teacher**				
Yes	144	19	1.96 (1.01, 3.83)	3 (1.03, 7.95)
No	85	22	1	1
**Health workers**				
Yes	168	21	2.62 (1.33, 5.17)	*
No	129	20	2.24 (1.13, 4.45)	
**Number of sexual partner**				
One	200	34	4.02 (2.19, 7.34)	*
Two and more	41	28	1	
**Amount of sexual experience**				
Once	20	28	1	
More than once with same sexual partner	201	22	12.8 (6.23, 26.39)	*
More than once with different sexual partner	20	12	2.33 (0.93, 5.84	
**Having romantic sexual relationship**				
Yes	233	36	21.04 (8.84, 50.04)	6.5 (1.23, 34.59)
No	8	26	1	1
**Duration of last sexual relationship**				
1-6 months	44	41	1	1
>6 months	197	21	8.74 (4.71, 16.24)	3 (1.02, 8.24)

*Not significant in the multivariate analysis (back ward stepwise logistic regression).

### Factors associated with voluntary counseling and testing service utilization

On bivariate analysis, the factors that were found to be significantly associated with VCT service utilization were sex, age, schooling attendance, educational status, mother’s educational status, co-residence with both parents, parental monitoring, parental communication, discussion of VCT services, having ever had a sexual partner, having ever had a sexual experience and perception of risk towards HIV/AIDS.

Out of variables which were entered to multiple logistic regression, sex, schooling attendance, educational status, co-residence with both parents, parental communication, discussion of VCT services with peer group and health workers, sexual experience and perception of risk towards HIV/AIDS were found to be significantly associated with VCT service utilization.

Female adolescents were about 2.6 times (AOR = 2.6, 95% CI: 1.79, 3.80) more likely to utilize VCT service than the males. The odds of having VCT was about 2 times (AOR = 2, 95% CI: 1.09, 3.41) higher for in school adolescents than out of school ones. Similarly, adolescents with secondary education and above were about 3 times more likely to use VCT as compared to those who have no formal education (AOR = 3, 95% CI: 1.11, 6.78).

Participants living with both parents were about 1.5 times more likely to use VCT services (AOR= 1.5, 95% CI: 1.02, 2.12). Adolescents who had had parental discussion on VCT services were 10times (AOR = 10, 95% CI: 6.09, 17.55) more likely to utilize the service as compared to those who had no parental communication. Discussion with peer groups/friends and health workers were the other variables associated with the utilization of VCT services.

Adolescents who had ever had sexual intercourse were about 4 times (AOR = 4, 95% CI: 2.09, 6.89) more likely to use VCT services than abstainers. Perceiving a risk towards HIV/AIDS was also found to be one of the predictors for the utilization of VCT. Adolescents who had a perception of risk towards HIV/AIDS were about 30 times more likely to utilize VCT services than those who had no perception of risk at all (AOR = 30, 95% CI: 10.65, 83.01) (Table 4).

**Table 4:** **Bivariate and multivariate analysis of factors associated with VCT service utilization among adolescents**, **Gondar town**, **Northwest Ethiopia**, **April 2012** (**n**=**1290**)

**Variables**	**VCT service utilization**		**Crude OR (95% CI)**	**Adjusted OR (95% CI)**
	**Yes**	**No**		
**Sex**				
Male	414	226	1	1
Female	519	131	2.16 (1.68, 2.78)	2.6 (1.79, 3.80)
**Age**				
15-16	338	198	1	**
17-19	595	159	2.19 (1.71, 2.81)	
**Schooling status**				
In school	779	288	1.24 (0.91, 1.69)	2 (1.09, 3.41)
Out of school	153	70	1	1
**Educational status**				
No formal education	30	37	1	
Primary education	347	192	2.23 (1.34, 3.72)	
Secondary education and above	555	129	5.31 (3.16, 8.91)	
**Maternal educational status**				
No formal education	482	217	1	**
Primary education	237	76	1.4 (1.04, 1.90)	
Secondary education and above	196	59	1.49 (1.07, 2.09)	
**Co residence with both parents**				
Yes	591	205	1.29 (1.01, 1.66)	
No	341	153	1	
**Parental monitoring**				
High	81	40	1	**
Low	851	318	1.32 (0.89, 1.97)	
**Parental communication**				
Yes	446	78	3.3 (2.49, 4.37)	10 (6.09, 17.55)
No	486	280	1	1
**Ever discussed about VCT service with Family/relatives**				
Yes	562	148	1.23 (0.93, 1.63)	**
No	331	107	1	
**Peer groups/friends**				
Yes	835	214	2.76 (1.79, 4.23)	3 (1.68, 4.89)
No	58	41	1	1
**Sexual partner**				
Yes	258	28	3.29 (2.17, 5.01)	**
No	635	227	1	
**Teacher**				
Yes	618	153	1.49 (1.12, 1.99)	**
No	275	102	1	
**Health workers**				
Yes	662	117	3.38 (2.53, 4.51)	4.5 (3.09, 6.51)
No	231	138	1	1
**Ever had sexual partner**				
Yes	379	66	3.03 (2.25, 4.08)	**
No	553	292	1	
**Ever had sexual experience**				
Yes	267	36	3.59 (2.47, 5.21)	4 (2.09, 6.89)
No	665	322	1	1
**Perception of risk towards HIV/AIDS**				
Yes	318	5	36.6 (14.97, 89.30)	30 (10.65, 83.01)
No	614	353	1	1

*Not significant in the multivariate analysis (back ward stepwise logistic regression).

## Discussion

The percentage of adolescents who attend health facilities for FP and HIV preventive services is one of the indicators of the immediate and long term RH needs of young people [11]. This community-based study which assessed the patterns of adolescent FP, VCT service utilization and associated factors is important to see the progress towards achieving the national MDGs, especially goals 4 to 6.

The study revealed that utilization of FP services was 79%; this finding was higher than those of studies conducted in Jimma (17.6%), Eastern parts of Gojam (21%) and Ghana (49%) [10,12,13]. The possible reason for the difference could be that the Jimma study consisted of both sexually experienced & inexperienced adolescents, whereas this study strictly focused on sexually experienced ones. Differences in the composition of the study subjects might be the reason for the different findings of Gojjam and Gondar; in Gojjam the study was conducted among out of school adolescents who were married and illiterate.

Experimentation with sex is a natural and normal part of adolescence, but experimentation without protection is one of the indicators of risky sexual behavior. This study revealed that contraceptive use at first sexual intercourse was only 68%. This finding showed how much these adolescents were at risk to different sexual and reproductive health problems, like unwanted pregnancy, unsafe abortion, STI’s and HIV/AIDS.

This study found that utilization of VCT service was much higher than a study conducted in Wollisso Woreda which was 5.3%, and in rural Butagira at 6% [14,15]. This is because the perception of risk of HIV/AIDS among sexually active adolescents was less than the results of this study. In addition, in the Wollisso area, there was low access to VCT service since there were only 3 facilities which provided VCT services. Moreover, lack of continuous supply of HIV testing re-agents and kits were the other problems which discouraged adolescents from utilizing VCT services. The difference in the findings of the Butagira study might be that the Butagira study was predominantly conducted among rural youth, in which the majority were out of school and had high levels of illiteracy. This in turn could be due to the fact that youth in rural areas have low access to information and VCT services.

In order to reach the UNAIDS goal of a 30 percent reduction in new HIV infections among young people by 2015, one of the measurable results is increasing the number of young people who know their status through counseling and testing services. Besides, bolstering these services and sustaining them among young people will be crucial in achieving “zero new HIV infections, zero discrimination and zero AIDS-related deaths”. However, this study revealed that the utilization of VCT among sexually experienced adolescents was only 88.4%.

In this study, the overall utilization of VCT services among males and females was 65% and 80%, respectively. These findings were higher than that of a study conducted in South Africa which was 9.7% for males and 10.9% for females [16]. The South Africa study was conducted in the rural areas of Eastern Cape Province, where HIV rapid tests were available at a small minority (approximately 10%) of clinics. According to the 2002 facility survey, HIV/AIDS counseling was routinely available in the rural areas of South Africa, but access to testing procedures was less common.

A substantial number of studies identified that adolescents with secondary education and above were more likely to utilize FP and VCT service [15-20]. This study also supported the above claim. This can be explained by the fact that educated adolescents have an increased knowledge about the availability of the service, the benefit of preventive health care, and have a higher receptivity towards new health-related information and better communication with their sexual partners.

Educated mothers are more open to discuss FP issues with their children. They are also more flexible to deal with problems faced by their children regarding reproductive health service utilization. Secondary and above educational attainment of the mother was also one of the factors significantly associated with FP service utilization. This finding is in line with a study which was conducted in East Gojam [12]. In addition, this research found out that discussion of the service with sexual partners, family/relatives, peer groups/friends and teachers had a significant association with FP service utilization. This can be justified by the fact that discussion of services with different categories of people allows adolescents to create more opportunities to exchange information, experiences, and build comprehensive knowledge about FP. It can also create opportunities to deal with adolescent problems associated with FP service utilization. Adolescents who have had romantic and lengthy sexual relationship were more likely to utilize FP service. This finding is consistent with studies conducted in the USA [21,22]. This could be because adolescents who have had romantic and lengthy sexual relationships may have more time to plan and discuss methods of protection to use during sexual activity. They are also more likely to care about each other and try to alleviate problems they may be facing during sexual relationships.

Female adolescents were more likely to utilize VCT services as compared to male adolescents. This finding is different from that of a study conducted in South and North Nigeria [20]. A possible explanation can be that the factors which help facilitate the utilization of VCT in this study (like perception of risk, having parental communication and discussion about the service with different categories of people) were found to be much lower among male participants than female ones.

School connectedness is one of the protective factors for adolescents in the ecological model of adolescent health. This study is in favor of the above association. Schooling status creates an opportunity to engage with different health promotion programs, like school based VCT service programs and school clubs which allow the adolescents to obtain more new information and knowledge related to the service.

Adolescents who were living with both parents were more likely to utilize VCT as compared to those who did not. This finding is against a study conducted in the UK which revealed that those who were living with single parents were more likely to utilize the service than those who were living with both parents [23]. This might be because most of the adolescents in the UK have a higher level of parental monitoring than the subjects in this study. This in turn may hinder service utilization. Living with both parents increases the likelihood of communication and discussion with adolescents, as well as helping shape adolescent behavior.

In this study, discussions with parents, peer groups/friends and health workers were significantly associated with adolescents’ VCT service utilization. This finding is consistent with a study done in Zambia [24]. This might be due to the fact that social relationships may influence young people’s decisions regarding HIV testing. Moreover, adolescents are more likely to engage in healthy behaviors when they feel connected to their family and peers. Since peer groups/friends are composed of individuals within similar age ranges, it also creates an opportunity to share information and experiences regarding VCT service.

Adolescents who had ever had sexual intercourse were more likely to utilize the VCT service than abstainers. This finding is in line with two studies conducted in Ndola, Zambia [24,25].

Perceiving a risk towards HIV/AIDS was also found to be a strong predictor of utilization of the service. This finding is similar with studies conducted in Nigeria and Zambia. These studies revealed that those perceiving a risk towards HIV were more likely to go for VCT than those who believed they were not at risk of contracting HIV [22,24]. This might be due to the fact that adolescents will not seek healthcare, like being tested for HIV, unless they view themselves as potentially vulnerable. Therefore, perceiving a risk towards HIV/AIDS is a cue to seeking healthcare, like knowing one’s own HIV status.

Generalization of the findings presented should be made with caution because of the following limitations: this study shared the limitations of other cross sectional studies, i.e. the difficulty of determining causal relationship between variables. Social desirability bias may have resulted in underreporting of family planning, voluntary counseling and testing utilization. Recall bias may also affect responses about events in some of the responses.

## Conclusions

In conclusion, the majority of adolescents were utilizing FP and VCT services in Northwest Ethiopia. But among sexually experienced adolescents, utilization of FP services at first sexual intercourse and VCT services were found to be low. The major reasons for not using family planning services were fear or embarrassment, followed by judgmental attitudes of health workers. In addition, partner or self-trust and embarrassment to use services were the major reasons for not using VCT. Thus, it is suggested that there be an intensified effort to increase the health service utilization for RH by adolescents, as they are the prime victims of a range of RH problems. Building life skills, facilitating parent to child communication, establishing and strengthening of youth centers and school reproductive health clubs are important steps to improve adolescent reproductive health service utilization.

## Competing interests

The authors declare that they have no competing interests.

## Authors’ contributions

SAF wrote the proposal, participated in data collection, analyzed the data and drafted the paper. ZBM, AFD and DNK approved the proposal with great revisions, participated in data analysis and revised subsequent drafts of the paper. All authors read and approved the final manuscript.

## Pre-publication history

The pre-publication history for this paper can be accessed here:

http://www.biomedcentral.com/1472-6963/13/294/prepub
